# HIV-1 transcription dominates over host gene activity at the HIV-1 integration site

**DOI:** 10.1128/mbio.02755-25

**Published:** 2025-11-24

**Authors:** Samuel Weissman, Yang-Hui J. Yeh, Miriam Viazmenski, Rachel Kim, Jack A. Collora, Savannah Steinhauser, Ya-Chi Ho

**Affiliations:** 1 Department of Microbial Pathogenesis, Yale University School of Medicine, New Haven, Connecticut, USA; The University of North Carolina at Chapel Hill School of Medicine, Chapel Hill, North Carolina, USA

**Keywords:** HIV-1 integration site, transcriptional interference, CRISPR activation and inhibition, insertional mutagenesis, HIV cure

## Abstract

Despite effective antiretroviral therapy (ART), HIV-1 persists as an integrated DNA provirus in the genome of infected cells. Host cells can regulate HIV-1 transcription at the HIV-1 integration site dependent on the location (actively transcribed genes vs repressive chromatin) and the orientation (in the same vs opposite orientation of the host gene transcription) of HIV-1 integration. Presumably, HIV-1 follows the host gene transcriptional activity at the HIV-1 integration site. We interrogated HIV-1-host gene transcriptional interactions at the HIV-1 integration site using CRISPR-mediated activation and inhibition of the host genes (in which HIV-1 was integrated) in seven HIV-1-infected Jurkat T cell clones with known HIV-1 integration sites in the introns of actively transcribed genes and a non-genic region. Using ATAC-seq and strand-specific RNA-seq to examine chromatin accessibility and RNA transcription levels, we found that host gene activation did not increase HIV-1 transcription, while host gene inhibition did not decrease HIV-1 transcription. HIV-1 drove high levels of aberrant host RNA transcription regardless of the HIV-1 integration orientation. HIV-1-driven aberrant host RNA transcription was inhibited by CRISPR-mediated HIV-1 inhibition but not by CRISPR-mediated host gene activation or inhibition. When HIV-1 was integrated into a non-genic region, HIV-1 increased host chromatin accessibility and drove high levels of aberrant host RNA transcription. Overall, HIV-1 LTR promoter-driven transcriptional activity dominated over the host promoter activity. HIV-1 transcription does not passively follow host gene activity. Our results highlight that despite effective ART, silencing HIV-1 promoter is required to inhibit HIV-1-driven aberrant host gene expression and chronic immune activation.

**IMPORTANCE:**

HIV-1 persists as an integrated provirus in infected cells. Antiretroviral therapy (ART) does not inhibit HIV-1 promoter activity. Therefore, despite effective ART, HIV-1 promoter continues to drive HIV-1 antigen expression and induce chronic immune activation. HIV-1 eradication relies on either effective HIV-1 latency reversal (the shock-and-kill strategy) or permanent HIV-1 silencing (the block-and-lock strategy). Therefore, understanding the transcriptional regulation of HIV-1 expression is key to HIV cure. Presumably, HIV-1 transcription passively follows host gene activity at the HIV-1 integration site. Using a CRISPR-mediated host gene activation and inhibition, we found that host gene activation does not increase HIV-1 transcription, while host gene repression does not inhibit HIV-1 transcription. HIV-1 drives aberrant host RNA expression even when HIV-1 is integrated into a non-genic region. Overall, HIV-1 dominates over host gene activity at the HIV-1 integration site. Despite ART, additional strategies silencing HIV-1 promoter activity are required to halt HIV-1-induced chronic immune activation.

## INTRODUCTION

Despite effective antiretroviral therapy (ART), HIV-1 persists lifelong as integrated proviral DNA in the genome of infected cells (1–3). Although ART blocks HIV-1 protein function and suppresses HIV-1 viral load to clinically undetectable levels, ART does not suppress HIV-1 long terminal repeat (LTR) promoter activity (4). Thus, despite ART, HIV-1 proviruses can be transcriptionally active (5–7), produce viral proteins (8–10), and induce chronic immune activation (11). These transcriptionally active HIV-1-infected cells presumably should be recognized by immune effectors and eliminated. However, despite decades of ART, HIV-1-infected cells escape immune clearance and persist lifelong (12–14). Understanding and targeting HIV-1 transcription, such as reactivating HIV-1 from latency in the shock-and-kill strategy or permanently silencing HIV-1 transcription in the block-and-lock strategy, is essential to HIV-1 eradication.

Host cells can affect HIV-1 transcription both *in trans* and *in cis*. Upon antigen stimulation and subsequent T cell activation, transcription factors NF-κB (15), NFAT (16), and AP-1 (17) bind to HIV-1 LTR promoter and activate HIV-1 transcription *in trans*. Recent studies identified how the chromatin environment surrounding the HIV-1 integration site shapes HIV-1 transcription *in cis* (18–21). More than 90% of HIV-1 integration sites are located in the introns of actively transcribed genes (22, 23) because of LEDGF and CPSF6-mediated HIV-1 integration site preference (24). Over time, these HIV-1 proviruses integrated in the introns of actively transcribed genes undergo stochastic reactivation, leading to immune recognition and elimination of the infected cells. Therefore, after long-term ART, intact HIV-1 proviruses integrated into transcriptionally repressive regions, such as zinc finger (ZNF) genes (7, 25, 26), non-genic regions, or centromeres (7), are presumably transcriptionally silent and thus evade immune recognition and elimination. Given that HIV-1 integrated into transcriptionally active regions is more prone to reactivation and that HIV-1 integrated into transcriptionally repressive regions is more likely silent, it is recently proposed that HIV-1 transcription passively follows the host gene activity at the HIV-1 integration site (26).

HIV-1 can affect host gene transcription *in cis*. HIV-1 integration increases local chromatin accessibility (27), brings in exogenous enhancer and transcription factor binding sites (such as NF-κB, NFAT, and AP-1 binding sites within HIV-1 promoter), and increases enhancer looping and local 3D chromatin interactions (18). HIV-1 integration in the same orientation as host genes can drive aberrant host gene expression through aberrant HIV-1-to-host chimeric RNA splicing (4, 28, 29) and readthrough transcription (27, 30). HIV-1 integration in the opposite orientation as host genes may cause transcriptional interference through RNA polymerase II collision or readthrough transcription (31, 32).

In the arms race between HIV-1 and host transcriptional regulation at the HIV-1 integration site, we postulate that HIV-1 transcription does not passively follow host gene activity at the HIV-1 integration site. We aim to determine the impact of host gene activation versus inhibition on HIV-1 transcription and examine HIV-1-host gene transcriptional interactions at the HIV-1 integration site. Here, using CRISPR-mediated activation (CRISPRa) and inhibition (CRISPRi) of host genes (into which HIV-1 is integrated) and HIV-1, we determined the impact of the host gene activity on HIV-1 transcription versus the impact of HIV-1 activity on host gene transcription. In six HIV-1-infected Jurkat T cell clones harboring known HIV-1 integration sites in the same or opposite orientation as the host gene transcription, we found that HIV-1 transcription dominated over host gene activity at the HIV-1 integration site. In one HIV-1-infected Jurkat T cell clone harboring HIV-1 integration into a non-genic region, we found that HIV-1 increased chromatin accessibility and drove aberrant host RNA transcription. Overall, HIV-1 transcription does not passively follow host gene activity.

## RESULTS

### Examination of HIV-1-host transcriptional interactions during CRISPRa versus CRISPRi of host gene versus HIV-1

**Fig 1:**
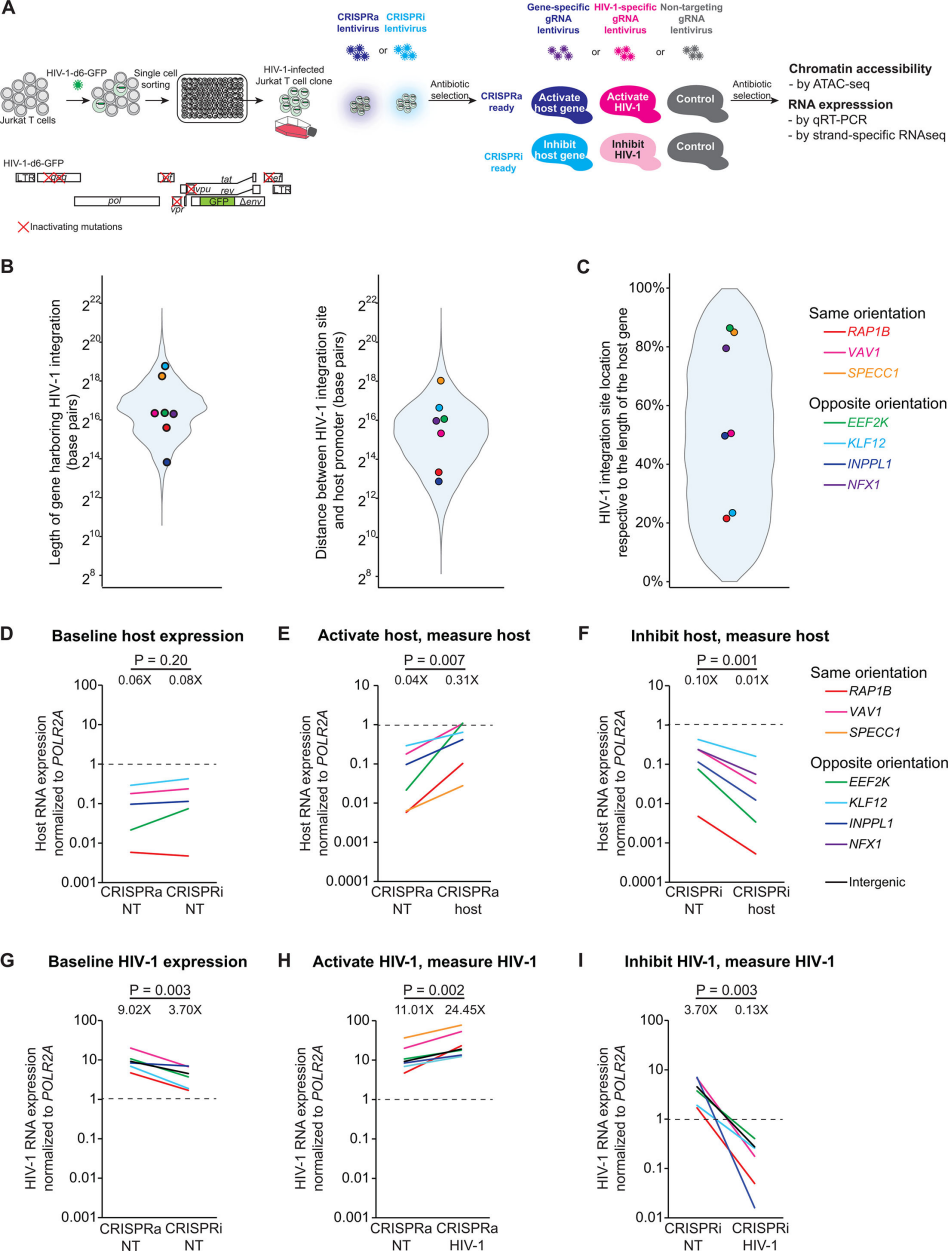
Experimental design. (**A**) Construction of CRISPRa- and CRISPRi-mediated host and HIV-1 activation and inhibition in HIV-1-infected Jurkat T cell clones. (**B**) Length of genes harboring HIV-1 integration and the location of HIV-1 integration sites in the HIV-1-infected Jurkat T cell clones in this study versus those identified from PLWH (38), represented as gray shades in the violin plot. (**C**) HIV-1 integration site locations relative to the gene harboring HIV-1 integration in the HIV-1-infected Jurkat T cell clones in this study versus those identified from PLWH (38). The distance from the HIV-1 integration site to the start of the transcription unit was divided by the length of the transcription unit. (**D–I**) Host gene and HIV-1 RNA expression at baseline (CRISPRa vs CRISPRi with nontargeting gRNA), after CRISPR activation, and after CRISPR inhibition. Host and HIV-1 RNA expression was measured by qRT-PCR normalized to housekeeping gene *POLR2A* expression (dashed lines). Geometric means of fold change are shown. *P* values were determined by paired two-tailed Student’s *t*-test of log-transformed values.

To examine HIV-1-host transcriptional interactions at the HIV-1 integration site, we constructed seven HIV-1-infected Jurkat T cell clones harboring a single-round HIV-1 reporter provirus with known HIV-1 integration sites (Table 1), as previously described (4, 18, 28, 33) (Fig. 1). Unlike JLat HIV-1-infected Jurkat T cell clones (34), which have minimal baseline HIV-1 expression, our HIV-1-infected Jurkat T cell clones are transcriptionally active (HIV-1-driven green fluorescent protein [GFP] expression) (4, 33) and thus provide a robust dynamic range to measure the impact of CRISPR-mediated perturbation on HIV-1-host transcriptional interactions. Briefly, Jurkat T cells were infected with an NL4-3-dE-EGFP-based single-round HIV-1 reporter virus (HIV-1-d6-dsGFP) (35) at a low multiplicity of infection to reduce multiple HIV-1 integrations within the same cell. This reporter virus contains inactivating point mutations in viral proteins to reduce viral cytopathic effects but preserves HIV-1 LTR promoter, Tat, Rev, and all HIV-1 *cis*-regulatory elements such as splice donor and acceptor sites, packaging signal (ψ), trans-activation responsive element (TAR), and Rev response element (RRE) that recapitulate HIV-1-host transcriptional interactions. Three days post-infection, GFP+ Jurkat T cells were sorted by flow cytometry as one cell per well in 96-well plates and cultured for 2 months into individual HIV-1-infected Jurkat T cell clones. HIV-1 integration site in each clone was determined by inverse PCR (23, 36). Overall, we constructed HIV-1-infected Jurkat T cell clones with eight integration sites. Three clones have HIV-1 integration in the same orientation as the host gene (*VAV1* for HIV-1-infected Jurkat T cell clone 8B10, *RAP1B* for clone 1G2, and *SPECC1* for clone 1D7), four have HIV-1 integration in the opposite orientation as the host gene (*EEF2K* for clone 1A8, *KLF12* for clone 2F5, *NFX1* for clone 8B10 which harbors two HIV-1 integration sites, and *INPPL1* for clone 5F9), and one has HIV-1 integration into a non-genic region (clone 1D9, with no genes within 15 kb of the HIV-1 integration site). HIV-1 integration into these host genes has been previously reported in people living with HIV (PLWH) (37). In these HIV-1-infected Jurkat T cell clones, the length of these host genes, the distance between the HIV-1 integration site and the host gene promoter (Fig. 1B), and the HIV-1 integration site location within the host genes (Fig. 1C) reflect the diverse HIV-1 integration sites in PLWH (38).

**TABLE 1:** Integration sites of HIV-1-d6-GFP-infected Jurkat T cell clones

Jurkat T cell clone	Gene	Orientation	Size of the transcription unit (bp)	HIV-1 integration site relative to transcription start site (bp)	HIV-1 integration site relative to the length of the transcription unit	HIV-1 integration site (hg38)	ENSEMBL transcript	Reported *in vivo*
1G2	*RAP1B*	Same	49,665	10,854	22%	chr12:68621712	ENST00000393436.9	Yes
8B10	*VAV1*	Same	84,654	41,940	50%	chr19:6814648	ENST00000602142.6	Yes
1D7	*SPECC1*	Same	309,668	262,952	85%	chr17:20272311	ENST00000395527.9	Yes
1A8	*EEF2K*	Opposite	82,461	71,284	86%	chr16:22277562	ENST00000263026.10	Yes
2F5	*KLF12*	Opposite	447,840	105,176	23%	chr13:74028753	ENST00000377669.7	Yes
5F9	*INPPL1*	Opposite	14,381	7,162	50%	chr11:72231929	ENST00000298229.7	Yes
8B10	*NFX1*	Opposite	80,642	64,106	79%	chr9:33354622	ENST00000379540.8	Yes
1D9	Non-genic					chr9:21093788		

These HIV-1-infected Jurkat T cell clones harboring known HIV-1 integration sites were individually and stably transduced with CRISPRa lentiviruses (dCas9-VP64 and MS2-p65-HSF1 transcription activator) (39) versus CRISPRi lentiviruses (dCas9-KRAB transcription repressor) (40) (Fig. 1A). Each of the HIV-1-infected, dCas9-transduced Jurkat T cell clones was further transduced with gRNAs to generate CRISPRa and CRISPRi of host gene, HIV-1, and nontargeting control, respectively. CRISPR-mediated host gene activation versus inhibition was measured using qRT-PCR of host gene RNA expression normalized to a housekeeping gene *POLR2A*. One gRNA per gene (out of three gRNAs tested for each gene), which had the most effective host gene activation (for CRISPRa) versus inhibition (for CRISPRi), was selected for further experiments (Table S1). Cell line clones that did not reach statistical significance in host gene activation or inhibition (CRISPRa of *NFX1* in clone 8B10 and CRISPRi of *SPECC1* in clone 1D7) were excluded from analysis. Of note, since the cell line clone 8B10 harbors 2 HIV-1 integration sites (into *VAV1* in the same orientation and *NFX1* in the opposite orientation), gRNAs perturbing HIV-1 in this clone were expected to activate proviruses integrated into both *VAV1* and *NFX1* and might thus play a role in modulating the global host gene expression pattern.

### HIV-1 LTR promoter drives high levels of HIV-1 RNA expression

We first measured host RNA expression at baseline and after CRISPR perturbations using qRT-PCR. The baseline host RNA expression (harboring HIV-1 integration) was lower than that of the housekeeping gene *POLR2A* (geometric mean 7%, range 0.5–43%) (Fig. 1D). The baseline host RNA expression was comparable in CRISPRa-nontargeting versus CRISPRi-nontargeting controls (6% vs 8% of *POLR2A*, *P* = 0.20)(Fig. 1D). CRISPRa of the host gene increased host RNA expression (7.8× increase relative to nontargeting control, *P* = 0.007) (Fig. 1E), while CRISPRi of the host gene reduced host RNA expression (12% relative to nontargeting control, *P* = 0.001)(Fig. 1F).

We next measured HIV-1 RNA expression at baseline and after CRISPR perturbations using qRT-PCR. The baseline HIV-1 RNA expression was higher than that of the housekeeping gene *POLR2A* (geometric mean 5.8×, range 1.7×–20×)(Fig. 1G). The baseline HIV-1 RNA expression was higher in CRISPRa-nontargeting control than that in CRISPRi-nontargeting control (geometric mean 2.4×, *P* = 0.003) (Fig. 1G). Of note, in the CRISPRa system, the p65 within the MS2-p65-HSF1 activator contains the transactivation domain of NF-κB p65 but not the DNA binding domain, and thus does not bind to HIV-1 promoter. In the CRISPRi system, the KRAB inhibitory domain inhibits HIV-1 expression only in the presence of HIV-1-targeting gRNA (41). There was no sequence homology between nontargeting gRNA and the HIV-1 reporter virus. We therefore normalized CRISPRa and CRISPRi effects of host and HIV-1 perturbations to respective CRISPRa-nontargeting and CRISPRi-nontargeting controls to account for the impact of CRISPRa and CRISPRi on baseline HIV-1 expression. CRISPRa increased HIV-1 RNA expression (2.2× relative to CRISPRa-nontargeting control, *P* = 0.002) (Fig. 1H), while CRISPRi reduced HIV-1 RNA expression (3% relative to CRISPRi-nontargeting control, *P* = 0.003) (Fig. 1I).

By comparing host RNA versus HIV-1 RNA expression, we found that HIV-1 RNA expression was substantially higher than host RNA expression at baseline (156.3× higher [range 23.6×–807.1×] in HIV-1 RNA than host RNA in CRISPRa-nontargeting control, *P* = 0.001; 42.6× higher [range 4.4×–359.0×] in HIV-1 RNA than host RNA in CRISPRi-nontargeting control, *P* = 0.006) (Fig. 1D and G). CRISPRi-mediated HIV-1 inhibition suppressed HIV-1 RNA level (13% relative to *POLR2A*) to that of baseline host RNA level (10% relative to *POLR2A*) (*P* = 0.75) (Fig. 1F and I). Taken together, we found that HIV-1 LTR promoter drove HIV-1 RNA expression higher than the host gene RNA expression and the housekeeping gene *POLR2A* RNA expression.

### HIV-1 transcription does not follow host gene activity

**Fig 2:**
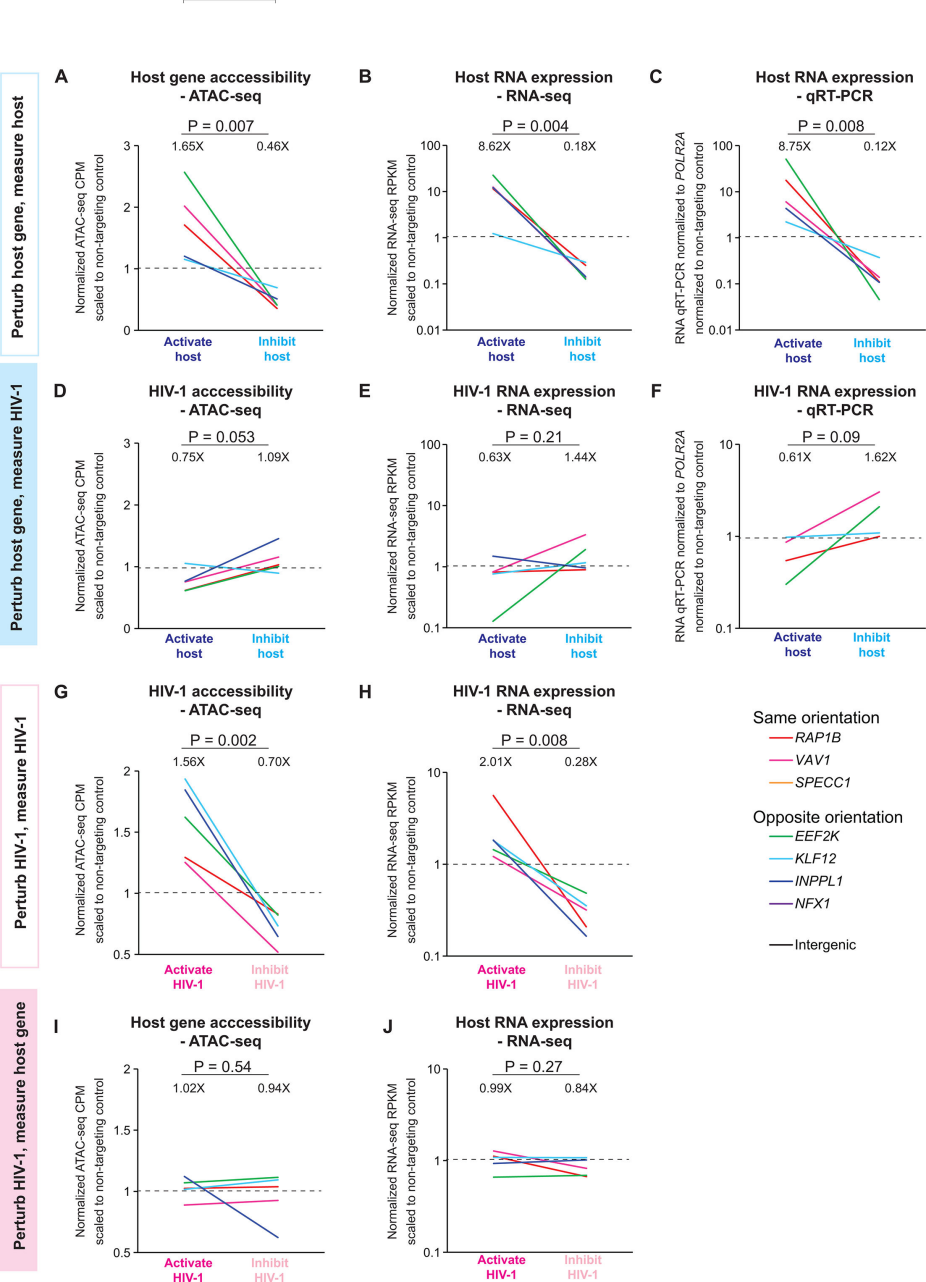
Impact of host gene perturbation on HIV-1 versus the impact of HIV-1 perturbation on host gene. (**A–C**) Impact of CRISPR-mediated host gene activation and inhibition on host gene chromatin accessibility and host RNA expression demonstrating effective CRISPR perturbation. (**D–F**) Impact of CRISPR-mediated host gene activation and inhibition on HIV-1 chromatin accessibility and HIV-1 RNA expression. (**G–H**) Impact of CRISPR-mediated HIV-1 activation and inhibition on HIV-1 chromatin accessibility and HIV-1 RNA expression demonstrating effective CRISPR perturbation. (**I–J**) Impact of CRISPR-mediated HIV-1 activation and inhibition on host chromatin accessibility and host RNA expression. Normalized ATAC-seq reads (counts per million reads, CPM) and RNA-seq reads (reads per kilobase per million reads, RPKM) were divided by corresponding ATAC-seq and RNA-seq reads from clones with CRISPRa-nontargeting and CRISPRi-nontargeting gRNA. Geometric means of fold change are shown. *P* values were determined by paired two-tailed Student’s *t*-test of log-transformed values.

To examine HIV-1-host transcriptional interactions at the HIV-1 integration site, we used triplicate measurements of ATAC-seq and RNA-seq for each CRISPR-perturbed HIV-1-infected Jurkat T cell clone (Fig. S1 to S4). As expected, CRISPRa of the host gene increased host gene chromatin accessibility, while CRISPRi of the host gene decreased host gene accessibility (geometric mean 1.65× increase in CRISPRa-host gene versus reduction to 46% in CRISPRi-host gene, *P* = 0.007) (Fig. 2A). Similarly, CRISPRa of the host gene increased host RNA expression measured by RNA-seq, while CRISPRi of the host gene decreased host RNA expression measured by RNA-seq (8.62× increase in CRISPRa-host gene vs reduction to 18% in CRISPRi-host gene, *P* = 0.004) (Fig. 2B). The CRISPR-induced host gene activation and inhibition level was consistent in qRT-PCR measurements (Fig. 2C). Surprisingly, we found that HIV-1 chromatin accessibility and HIV-1 RNA expression did not follow the activation and inhibition of the host gene. CRISPRa of the host gene modestly decreased HIV-1 chromatin accessibility, while CRISPRi of the host gene modestly increased HIV-1 accessibility (reduction to 75% in CRISPRa-host gene vs 1.09× increase in CRISPRi-host gene, *P* = 0.053) (Fig. 2D). Similarly, CRISPRa of the host gene modestly decreased HIV-1 RNA expression measured by RNA-seq, while CRISPRi of the host gene modestly increased HIV-1 RNA expression measured by RNA-seq (reduction to 63% in CRISPRa-host gene vs 1.44× increase in CRISPRi-host gene, *P* = 0.21)(Fig. 2E), consistent with qRT-PCR measurements (Fig. 2F). Of note, this trend did not reach statistical significance, with heterogeneous integration site-dependent differences between clones. Yet, host gene activation did not increase HIV-1 RNA expression, while host gene inhibition did not decrease HIV-1 RNA expression. Overall, we found that HIV-1 transcription did not follow the activation and inhibition of the host gene activity.

### HIV-1 transcription does not affect overall host gene activity

We next examined the impact of HIV-1 activation versus inhibition on host gene activity. As expected, CRISPRa of HIV-1 increased HIV-1 chromatin accessibility, while CRISPRi of HIV-1 decreased HIV-1 accessibility (1.56X increase in CRISPRa-HIV-1 versus reduction to 70% in CRISPRi-HIV-1, *P* = 0.002)(Fig. 2G). Similarly, CRISPRa of HIV-1 increased HIV-1 RNA expression measured by RNA-seq, while CRISPRi of HIV-1 decreased HIV-1 RNA expression measured by RNA-seq (2.01X increase in CRISPRa-HIV-1 vs reduction to 28% in CRISPRi-HIV-1, *P* = 0.008)(Fig. 2H). HIV-1 activation versus inhibition did not significantly change host gene chromatin accessibility and host RNA expression (Fig. 2I and J).

### HIV-1 drives high levels of aberrant host RNA expression that cannot be suppressed by host gene activation or inhibition

**Fig 3:**
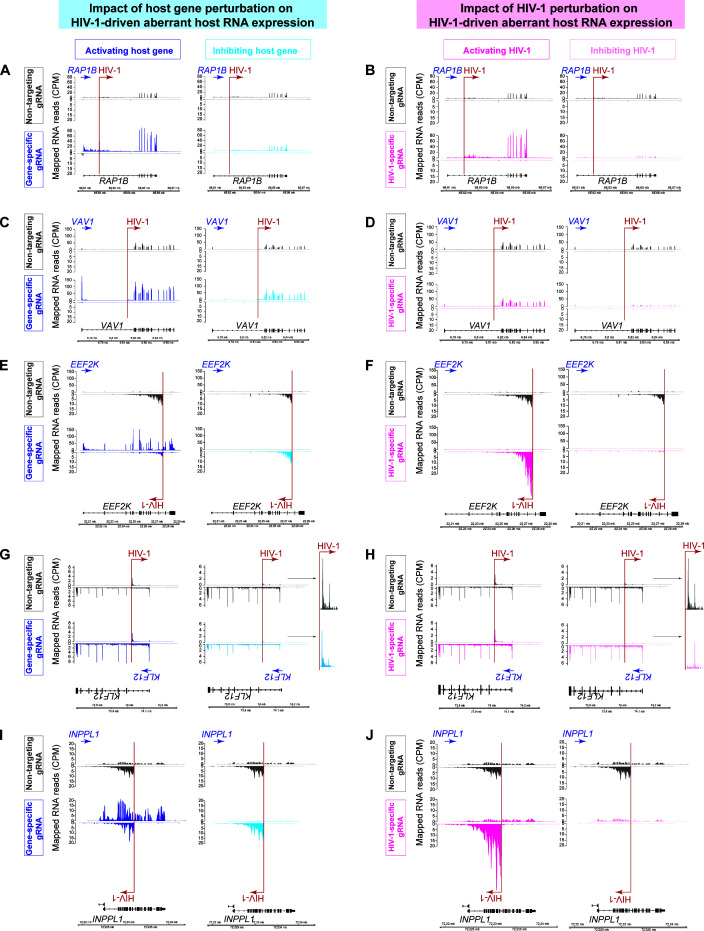
HIV-1-host transcriptional interference at the HIV-1 integration site. Mapped RNA-seq reads (counts per million, CPM) by strand-specific RNA-seq at HIV-1 integration sites in the same orientation [*RAP1B* (**A–B**), *VAV1* (**C–D**)] and the opposite orientation [*EEF2K* (**E–F**), *KLF12* (**G–H**), *INPPL1* (**I–J**)] upon activation and inhibition of host versus HIV-1.

HIV-1 can drive high levels of aberrant host gene expression downstream of the HIV-1 integration site through HIV-1-to-host aberrant RNA splicing (4, 28) or HIV-1 3′ LTR activity (27, 30). We postulated that averaging host RNA expression both upstream and downstream of the HIV-1 integration site failed to capture HIV-1-driven aberrant host RNA expression. We examined the host gene RNA-seq tracks at the HIV-1 integration sites (Fig. 3; Fig. S1 to S4). As we previously reported, when HIV-1 was integrated in the same orientation as the host gene, HIV-1 drove high levels of aberrant RNA expression downstream of the HIV-1 integration site (Fig. 3A through D). While CRISPRi of HIV-1 suppressed HIV-1-driven aberrant host RNA expression as expected (Fig. 3B and D), CRISPRi of the host gene failed to suppress HIV-1-driven aberrant host RNA expression (Fig. 3A and C). Whether HIV-1 integration into the opposite orientation drove aberrant host RNA expression was unknown. We found that when HIV-1 was integrated into the opposite orientation of the host gene, HIV-1 drove high levels of aberrant antisense host RNA expression (Fig. 3E through J). While CRISPRi of HIV-1 suppressed HIV-1-driven aberrant antisense host RNA expression (Fig. 3F, H and J), CRISPRa or CRISPRi of the host gene failed to suppress HIV-1-driven aberrant antisense host RNA expression (Fig. 3E, G and I). Overall, we found that HIV-1 drove high levels of aberrant host RNA expression that could not be suppressed by host gene activation or inhibition. HIV-1-driven aberrant host gene expression dominated over host gene activity.

### HIV-1 integration into a non-genic region increases host chromatin accessibility, maintains active HIV-1 transcription, and induces aberrant host RNA transcription

**Fig 4:**
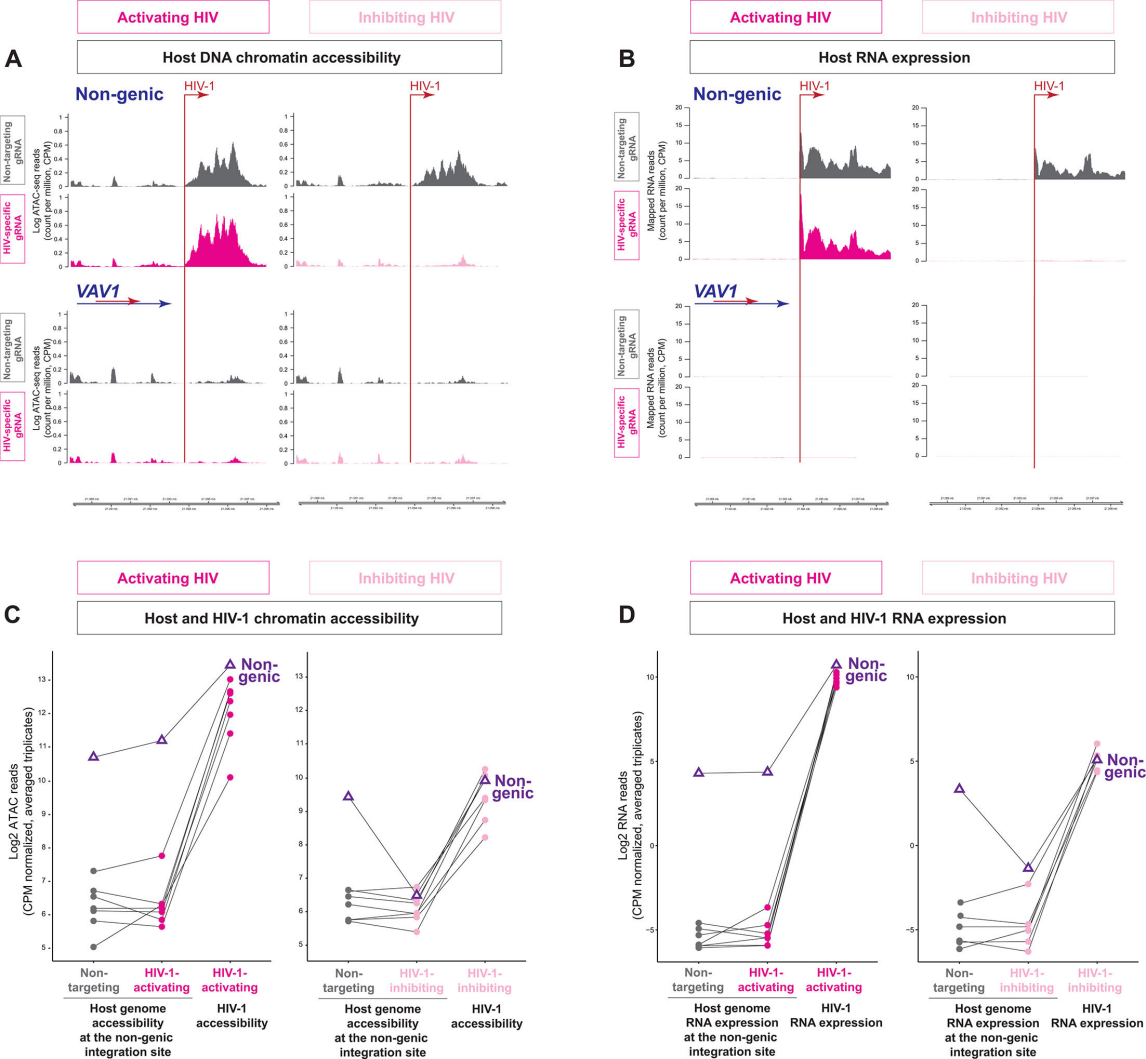
Impact of HIV-1 integration into a non-genic region on host chromatin accessibility and aberrant host RNA expression. ATAC-seq (**A**) and RNA-seq (**B**) of an HIV-1-infected Jurkat T cell clone 1D9 in which HIV-1 was integrated into a non-genic region, compared with another HIV-1-infected Jurkat T cell clone 8B10 in which HIV-1 was integrated into different sites (*VAV1* and *NFX1*), not at this non-genic region. (**C–D**) Host gene accessibility (**C**) and host RNA transcription (**D**) of non-genic region integration (1D9, purple triangles) versus other HIV-1-infected Jurkat T cell clones. Each dot represents one HIV-1-infected Jurkat T cell clone listed in Table 1.

Finally, we examined the impact of HIV-1 integration into a non-genic region of the host genome (Fig. 4). Compared with HIV-1-infected Jurkat T cell clones harboring HIV-1 integration into other regions (such as *VAV1*, Fig. 4A), we found that HIV-1 integration into a non-genic region induced aberrant increases in local host chromatin accessibility ~4,000 bp downstream of HIV-1 integration site (Fig. 4A, gray). HIV-1-induced aberrant increase in host chromatin accessibility was further increased upon HIV-1 activation and reduced upon HIV-1 inhibition (Fig. 4A). At the RNA transcription level, HIV-1 integration into the non-genic region induced high levels of aberrant host RNA transcription >4,000 bp downstream of the HIV-1 integration site, which was abrogated by HIV-1 inhibition (Fig. 4B). Compared with the other six HIV-1-infected Jurkat T cell clones (as listed in Table 1) harboring HIV-1 integration into host transcription units, we found that despite integration into a non-genic region, the HIV-1 genome was highly accessible (Fig. 4C) and HIV-1 RNA was highly expressed (Fig. 4D). Of note, this was the only HIV-1-infected Jurkat T cell clone harboring HIV-1 integration into a non-genic region. The impact of HIV-1 integration into other non-genic regions in a transcriptionally repressive environment, such as zinc-finger genes or centromere (7, 12), requires further investigation in respective cell line clones and primary cells. Overall, we found that despite integration into a non-genic region, HIV-1 integration increased host chromatin accessibility at the integration site, drove aberrant host RNA transcription, and maintained active HIV-1 transcription.

## DISCUSSION

Our study of eight HIV-1 integration sites in HIV-1-infected Jurkat T cell clones depicted HIV-1-host transcriptional interactions in three different HIV-1 integration scenarios: HIV-1 integration in the same orientation as the host gene, in the opposite orientation as the host gene, and in a non-genic region. Importantly, we used paired CRISPRa-mediated activation versus inhibition of host gene versus HIV-1 to mechanistically interrogate whether host gene activity affected HIV-1 transcription, and whether HIV-1 LTR promoter activity affected host gene activity. Our study identified that HIV-1 LTR promoter dominated over host gene activity at the HIV-1 integration site and remained transcriptionally active despite host gene repression. Regardless of the HIV-1 integration orientation, that is, in the same versus opposite orientation as the host transcription unit, HIV-1 drove high levels of aberrant host RNA expression. When integrated into a non-genic region, HIV-1 increased host chromatin accessibility and drove high levels of aberrant host RNA expression.

Of note, given the highly heterogeneous HIV-1 integration sites *in vivo* and the few HIV-1 integration sites examined in this study, further studies involving more HIV-1 integration sites and in primary cells would be needed for more generalizable conclusions. In addition, *in vitro* culture may not recapitulate *in vivo* clonal expansion dynamics. Notably, using a murine stem cell virus (a gammaretrovirus) retroviral cassette harboring splice donor and acceptor sites, the *in vivo* retroviral insertional mutagenesis screen recapitulated how HIV integration into *BACH2* drives clonal expansion, including retroviral insertion enrichment immediately upstream of *Bach2* translation start site, in the same orientation as the *Bach2* transcription unit, retroviral-host chimeric RNA, and striking *in vivo* clonal expansion of the transduced T cells (42). Whether HIV-1 integration site-driven clonal expansion and HIV-1-host interactions are influenced by tissue microenvironment, such as high BACH2 activity in human gut (43), remains to be determined.

Advances in HIV-1 integration site analysis (6, 7, 21, 26, 44, 45) showed that most HIV-1-infected cells harboring intact and inducible HIV-1 proviruses are eliminated by immune selection pressure after long-term ART. The remaining HIV-1-infected cells harboring intact HIV-1 proviruses survive immune selection pressure because of low transcriptional activity when integrated into transcriptionally repressive regions, such as ZNF genes and centromeres (6, 7, 12, 14, 26, 44, 45). By targeted insertion of HIV-1 reporter virus into ZNF genes, it was reported that HIV-1 integration passively followed local host gene activity (26). However, HIV-1 reporter systems lacking major HIV-1 splice sites for HIV-1-to-host RNA splicing (4, 27, 28, 46, 47), lacking introns that counteract HUSH silencing (48–50), and lacking HIV-1 3′ LTR that drives downstream host chromatin opening (27) and readthrough transcription (30) cannot recapitulate HIV-1-host interactions at the HIV-1 integration site.

Presumably, HIV-1 passively follows the host gene transcriptional activity at the HIV-1 integration site. We only found one out of seven cell line clones that followed this trend. In the HIV-1-infected Jurkat T cell clone 5F9, host gene (*INPPL1*) activation increased HIV-1 RNA transcription, while host gene inhibition decreased HIV-1 RNA transcription. Compared with HIV-1 integration sites observed in PLWH, the size of this gene *INPPL1* is small (Fig. 1B). HIV-1 (a 9.4 kb reporter provirus) was integrated into *INPPL1* (14.3 kb) within 7.2 kb of the host promoter. We speculate that the close proximity of two convergent promoters (*INPPL1* vs HIV-1) may create additional HIV-1-host transcriptional interaction. Examination of more clones having similar proximity of HIV-1 and host gene promoters is required to identify more generalizable results.

Human gene expression is tightly regulated at the epigenetic and transcriptional levels. Yet, retroviruses integrate into the host genome and transform the infected cells into cancer through insertional oncogenesis (51, 52). Gammaretroviruses, frequently used as a vector for gene therapy, may drive cancer through insertion into promoter regions of host genes (53, 54) or by insertional activation of oncogenes by the myeloproliferative sarcoma virus (MNDU3) promoter-enhancer (55). Lentiviruses, including HIV, integrate into the introns and usually do not drive cancer with rare exceptions (30). Caution is needed when using lentiviral vectors, which were previously considered safe in gene therapy compared to oncogenic gammaretroviral vectors. On the other hand, in chimeric antigen receptor (CAR) T cell treatment for cancer, integration of the lentiviral CAR cassette into specific cancer genes can potentiate CAR-T cell therapeutic effect by promoting the persistence of CAR T cells (by insertional activation of *BACH2*) (42) or driving proliferation of specific clones (by insertional disruption of *TET2* or insertional activation of *VAV1*) (56, 57). Thus, understanding how lentiviral integration changes host gene expression not only advances our understanding in HIV-1 persistence but also improves clinical applications involving lentiviral vectors, such as CAR T cell engineering and gene therapy.

## MATERIALS AND METHODS

### Construction of CRISPRa and CRISPRi HIV-1-infected Jurkat T cell clones

 HIV-1-infected Jurkat T cell clones having known HIV-1 integration sites were constructed, as previously described (18, 28, 33). The HIV-1-d6-dsGFP reporter lentivirus was constructed from the NL4-3 plasmid backbone by disrupting *env* with GFP insertion, truncating *nef*, and creating inactivating point mutations in *gag* before and after an internal ribosomal entry site in *gag* (M6* and M142*), *vif* (Y30*), *vpr* (Q8*), and *vpu* (I14X) through site-directed mutagenesis (35). This HIV-1 reporter expresses Tat and Rev and contains all HIV-1 *cis*-regulatory elements, such as splice donor and acceptor sites, ψ, TAR, and RRE that recapitulates HIV-1-host transcriptional interactions. HIV-1 integration site was determined by inverse PCR (23, 36).

Lentiviral vectors harboring CRISPR and gRNA cassettes were transfected along with pMDLg/pRRE (Addgene #12251)(58), pRSV-Rev (Addgene #12253), and pCMV-VSV-G (Addgene #8454) in Lenti-X HEK293T cells (Takara Bio, #632810) through Lipofectamine 2000 (Thermo Fisher Scientific, #11668027). Lentiviruses were concentrated through the Lenti-X concentrator (Takara #631231). HIV-1-infected Jurkat T cell clones were transduced with lentiviruses carrying dCas9 and gRNA cassettes sequentially and underwent antibiotic selection for at least one week. As a control, untransduced HIV-1-infected Jurkat T cell clones died within 1 week, demonstrating effective antibiotic selection.

For CRISPR-ready HIV-1-infected Jurkat T cell clones, HIV-1-infected Jurkat T cell clones (1G2, 8B10, 1D7, 1A8, 2F5, 5F9, and 1D9) were transduced with pseudotyped lentiviral vectors Lenti-MPHv2 (containing EF1α-driven MS2-p65-HSF1 activator and hygromycin selection marker, Addgene #89308) (39) for CRISPRa and Lenti-dCas9-KRAB-blast (containing blasticidin selection marker, Addgene #89567) (40) for CRISPRi, respectively. Two days after transduction, cells underwent 1 week of antibiotic selection (100 µg/mL hygromycin for CRISPRa and 8 µg/mL blasticidin for CRISPRi).

Then, to introduce gRNA, each HIV-1-infected, CRISPR-ready Jurkat T cell clone was transduced with gene-targeting, HIV-1-targeting, or nontargeting gRNA (Table S1). For CRISPRa, gRNAs were cloned into lentiSAMv2 (containing blasticidin selection marker, Addgene #75112) (39), which contains dCas9-VP64 and MS2 binding loop in the sgRNA backbone to recruit activation domains p65 and HSF1 (encoded on the Lenti-MPHv2 vector). This synergistic activation mediator (SAM) complex increases dCas9-VP64-mediated gRNA-specific activation. For CRISPRi, gRNAs were cloned into pCRISPRia-v2 (containing puromycin selection marker, Addgene #84832) (59). Two days after transduction, cells underwent antibiotic selection for at least 1 week with 100 µg/mL hygromycin and 8 µg/mL blasticidin for CRISPRa and gRNA-transduced HIV-1-infected Jurkat T cell clones and 8 µg/mL blasticidin and 1.5 µg/mL puromycin for CRISPRi and gRNA-transduced HIV-1-infected Jurkat T cell clones.

### qRT-PCR

The effect of CRISPRa-mediated host gene and HIV-1 activation and CRISPRi-mediated host gene and HIV-1 inhibition was examined by relative qRT-PCR by normalizing host gene and HIV-1 RNA to housekeeping gene *POLR2A* and CRISPR-nontargeting gRNA transduced cells. Briefly, triplicates of 50,000 cells were resuspended in TRIzol reagent (Thermo Fisher Scientific, #15596026) for RNA extraction (Direct-zol-96 RNA, Zymo Research #R2056). Relative qRT-PCR of the host genes and HIV-1 was performed using Thermo Fisher TaqMan Gene Expression Assays and qScript XLT 1-Step RT-qPCR ToughMix Low ROX (QuantaBio, # 95134-100) on a QuantStudio 3 Real-Time PCR system (Thermo Fisher Scientific). Primers and probes targeting host genes (FAM-MGB) (Table S1) and HIV-1 RNA (FAM-MGB) (11, 60) were separately duplexed with the housekeeping gene *POLR2A* (Hs00172187_m1, VIC-MGB).

### ATAC-seq and RNA-seq

To increase the quality of ATAC-seq and RNA-seq, cells from each Jurkat T cell clone underwent Ficoll density gradient centrifugation for dead cell removal (Ficoll-Paque PLUS Medium, VWR #95038-168). Approximately 50,000 cells per aliquot (three replicates per Jurkat T cell clone) were used for ATAC-seq and 50,000 cells per aliquot were used for RNA-seq. ATAC-seq was performed as we previously described (17). Briefly, aliquots of 50,000 cells were resuspended in 50 µL ATAC-lysis buffer (10 mM Tris-HCl at pH 7.4, 10 mM NaCl, 3 mM MgCl_2_, 0.1% Tween-20, 0.1% NP-40, and 0.01% digitonin) on ice for 3 min. We then diluted the mixture with 1 mL ATAC-wash (10 mM Tris-HCl at pH 7.4, 10 mM NaCl, 3 mM MgCl_2_, and 0.1% Tween-20). Cells were washed, pelleted, and transposed for 30 min at 37°C (1× TD buffer with 33% PBS, 0.1% Tween-20, 0.01% digitonin, and 2.5 µL TDE1). After transposition, DNA was isolated using Qiagen MinElute PCR cleanup kit (#28004). Library prep was performed using NEBNext Ultra II Q5 master mix (NEB #M0544X) and Nextera indexed according to NEBNext Ultra II (NEB #E7645) instructions. Following baseline amplification, typically eight cycles, SYBR qPCR was used to determine total cycle numbers needed. Typically, a total of 12 additional PCR cycles of library amplification were added, depending on qPCR results. After validating library size on an Agilent TapeStation, 150 bp paired-end sequencing was performed by NovaSeq 6000 at Yale Center for Genome Sequencing.

 For RNA-seq, triplicates of 50,000 cells were resuspended in TRIzol reagent (Thermo Fisher Scientific, #15596026) for RNA extraction (Direct-zol-96 RNA, Zymo Research #R2056). To identify sense versus antisense host gene and HIV-1 RNA transcription, we used NEBNext Ultra II Directional RNA Library Prep Kit for Illumina for RNA-sequencing (NEB #E7760) for library prep with NEBNext rRNA Depletion Kit v2 (NEB #E7400). cDNA libraries were sequenced on NovaSeq 6000.

### Bioinformatic analysis of ATAC-seq and RNA-seq

 To generate a reference genome for HIV-1 and the human genome, the HIV-1-d6-GFP sequence was added as a “25th chromosome” to the hg38 reference genome accessed from NCBI. Where relevant, we used the Gencode V44 basic gene annotations and added the HIV-1 annotations.

 For ATAC-seq and RNA-seq analysis, adaptor sequences were removed with Cutadapt (61) (v4). We proceeded with ATAC-seq analysis by mapping reads to our reference genome with Bowtie 2 (v2.5) with set parameters “--very-sensitive -k 20” (62). We called ATAC peaks with Genrich (https://github.com/jsh58/Genrich) arguments “-j -y -r -e chrM -v” as in reference 18 after SAMtools (63) (v1.17) was used to sort and index the mapped reads. Peaks were merged across all samples via BEDTools (64) (v2.30.0). Next, duplicate reads were identified with Picard’s (v3.0.0; https://broadinstitute.github.io/picard/; Broad Institute) MarkDuplicates function. For each set of samples to be directly compared (i.e., the gene-activating or gene-inhibiting samples for a given integration site), SAMtools was used to down-sample reads without replacement to the lowest molecular complexity among the compared samples. The Picard-identified duplicate reads were then removed. We quantified reads in peaks with BEDTools multicov and generated coverage data in a bigWig file at 10 nt resolution with the deepTools (v3.5) bamCoverage function (65).

For the RNA-seq analysis, mapping was done with the STAR aligner (v2.7.10b) on settings “--chimSegmentMin 20 --chimOutType WithinBAM” to identify splicing between HIV-1 and the human genome (66). Again, the reads were downsampled with SAMtools to give similar numbers of reads among the compared samples. Finally, the reads mapping to each gene were quantified with featureCounts. For the purposes of analysis, annotations were added corresponding to the groups of exons upstream and downstream of each analyzed genic HIV-1 integration site. We also added annotations of length 5 kb in either orientation of the HIV-1 integration site on the antisense strand of the gene of HIV-1 integration.

### Normalization, differential expression, and coverage analysis for RNA-seq and ATAC-seq

ATAC-seq data were normalized by the R function normOffsets in the csaw package (67) (Bioconductor package v3.17). This method designed for ATAC-seq uses a quantile regression to compute a normalization factor for each gene as a function of its expression level. RNA-seq data were normalized by TMM (68).

We generated coverage plots based on triplicate-averaged 10-nucleotide resolution coverage data from deepTools bamCoverage function (65). This was set to be strand-specific for our RNA-seq data. We then visualized the coverage using the R package Gviz (Bioconductor package v3.17) (69) (Fig. 3; Fig. S1 to S4).

Hypothesis testing for RNA-seq and ATAC-seq data were performed by pairwise comparisons between gene-targeting and nontargeting samples and between HIV-targeting and nontargeting samples. In general, triplicates were available for each of the two compared conditions. For both RNA-seq and ATAC-seq, we used the edgeR package’s quasi-likelihood method to determine genes and peaks that were differentially expressed (70). For ATAC-seq analysis, *P* values of nearby peaks were merged up to 10 kb using the lowest Bonferroni-adjusted *P* value in each set of merged peaks for optimal sensitivity. Calculated *P* values were then Bonferroni-adjusted for the six comparisons performed on each sample (Fig. S1 to S4).

## Data Availability

ATAC-seq and RNA-seq data have been deposited to GEO with accession numbers GSE302221 (ATAC-seq) and GSE302199 (RNA-seq). These will be publicly available upon publication.

## SUPPLEMENTAL MATERIAL

Supplemental material mbio.02755-25-s0001.pdf Table S1; Figures S1-S4.

ASM does not own the copyrights to Supplemental Material that may be linked to, or accessed through, an article. The authors have granted ASM a non-exclusive, world-wide license to publish the Supplemental Material files. Please contact the corresponding author directly for reuse.
