# Supplementary material for: HIV-1 transcription dominates over host gene activity at the HIV-1 integration site
Source: mBio. 2025 Nov 24;17(1):e02755-25. doi: 10.1128/mbio.02755-25 (PMC12802265; doi:10.1128/mbio.02755-25)
Supplement: Supplemental material — Table S1; Figures S1-S4. [file mbio.02755-25-s0001.pdf]

Supplemental material
Table S1. CRISPR gRNA sequences and qRT-PCR primer-probe sets

Figure S1. Impact of host versus HIV-1 perturbation on host versus HIV-1 chromatin accessibility when HIV-1 is integrated into host genes in the same orientation at the *RAP1B* and *VAV1* integration sites.

Figure S2. Impact of host versus HIV-1 perturbation on host versus HIV-1 chromatin accessibility in the HIV-1-infected Jurkat T cell clone 1D7 at the *SPECC1* integration site.

Figure S3. Impact of host versus HIV-1 perturbation on host versus HIV-1 chromatin accessibility when HIV-1 is integrated into host genes in the opposite orientation at the *EEF2K*, *KLF2*, and *INPPL1* integration sites.

Figure S4. Impact of host versus HIV-1 perturbation on host versus HIV-1 chromatin accessibility in the HIV-1-infected Jurkat T cell clone 8B10 at the *NFX1* integration site.

Table S1. CRISPR gRNA sequences and qRT-PCR primer-probe sets

Jurkat T cell clone	Target gene	CRISPRa gRNA target	CRISPRi gRNA target	qRT-PCR primer/probe set
1G2	*RAP1B*	ATGCAGGCCGGCTAGAGGC	GGGCTGAACGCCTGACGTCA	Hs04194442_s1
8B10	*VAV1*	GCCCAGGCCTGTGTCGAGT	GGTCGCTCCACAGGCGAGCA	Hs01041599_m1
	NFX1	N/A	GTAGGTTCTGCGGCACGGGA	Hs01048860_m1
1D7	*SPECC1*	TACACTGGAGGTTTTCTAA	N/A	Hs01060503_m1
1A8	*EEF2K*	CAGCGAGGGGGCAATTCGA	GACAGCGAGCGGGACTCGGG	Hs01108504_m1
2F5	*KLF12*	AGCCCGCCTCCTAATCCTC	GAGCCCGCCTCCTAATCCTC	Hs00971556_m1
5F9	*INPPL1*	GCGGCGCGGGGTTGAGCGT	GGGCTTGAGGATCCGTGGGC	Hs01071824_g1
	HIV-1	CTACAAGGGACTTTCCGCTG	CTACAAGGGACTTTCCGCTG	
	Nontargeting (NT)	GTGCACCCGGCTAGGACCGG	GTGCACCCGGCTAGGACCGG	

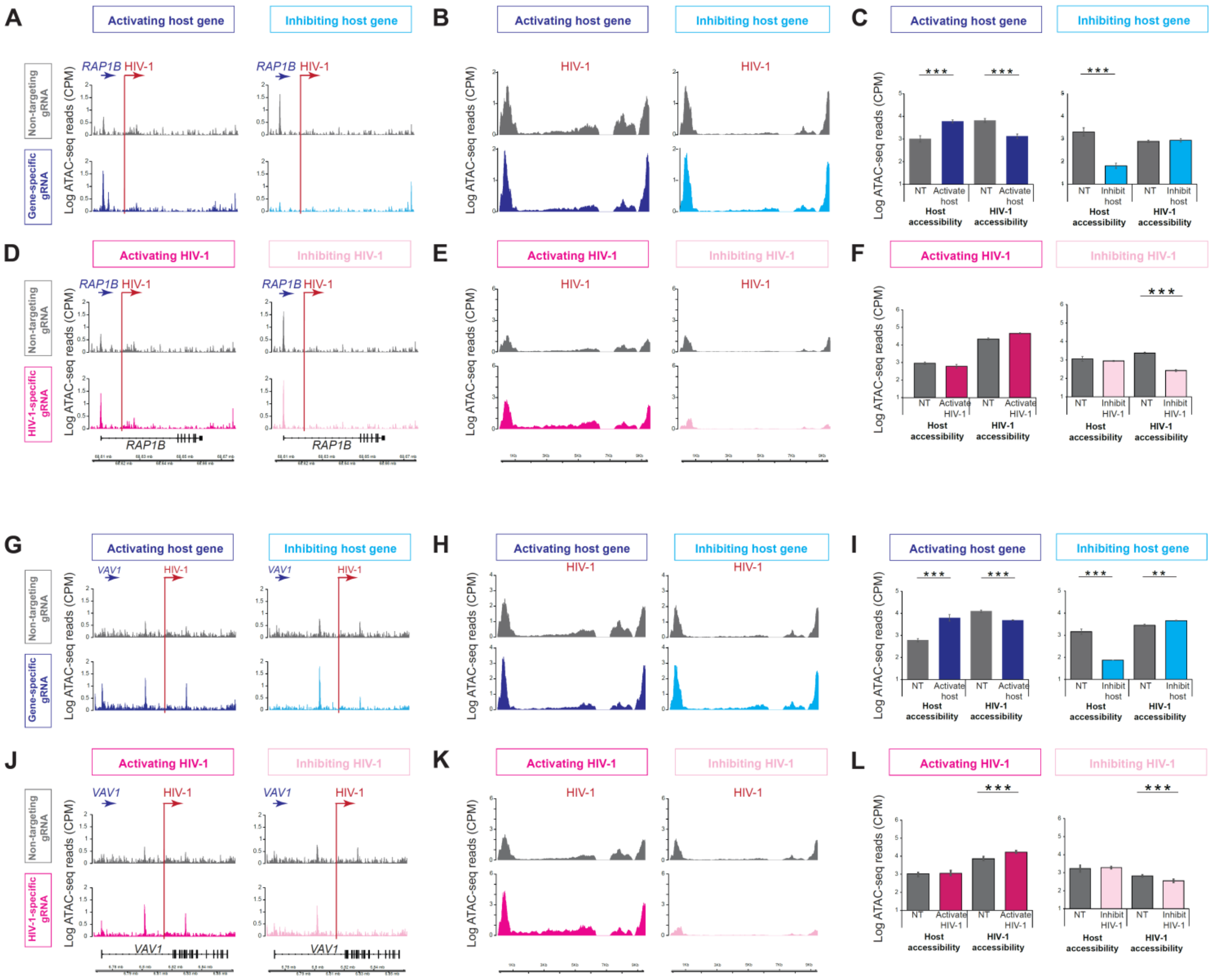

Figure S1. Impact of host versus HIV-1 perturbation on host versus HIV-1 chromatin accessibility when HIV-1 is integrated into host genes in the same orientation at the *RAP1B* and *VAV1* integration sites. (**A**–**L**) Host gene and HIV-1 chromatin accessibility upon CRISPR activation versus inhibition of host gene versus HIV-1. Host gene and HIV-1 chromatin accessibility after activation versus inhibition of the host gene (**A**–**C, G**–**I**) and HIV-1 (**D**–**F, J**–**L**) in Jurkat T cell clones harboring HIV-1 integrated into *RAP1B* (**A**–**F**) and *VAV1* (**G**–**L**). For ATAC-seq analysis, to account for patterns of chromatin accessibility, peaks within 10 kb were assigned a single Bonferroni-corrected P value equal to the lowest P value among the peaks, divided by the number of peaks within the 10 kb region. Calculated P values were then Bonferroni-adjusted for the six comparisons performed on each sample determined by edgeR. * $P < 0.05$; ** $P < 0.01$; *** $P < 0.001$. Of note, HIV-1 ATAC-seq and RNA-seq reads were mapped to both HIV-1 5’ LTR and 3’ LTR because of the identical sequence in the LTR repeats.

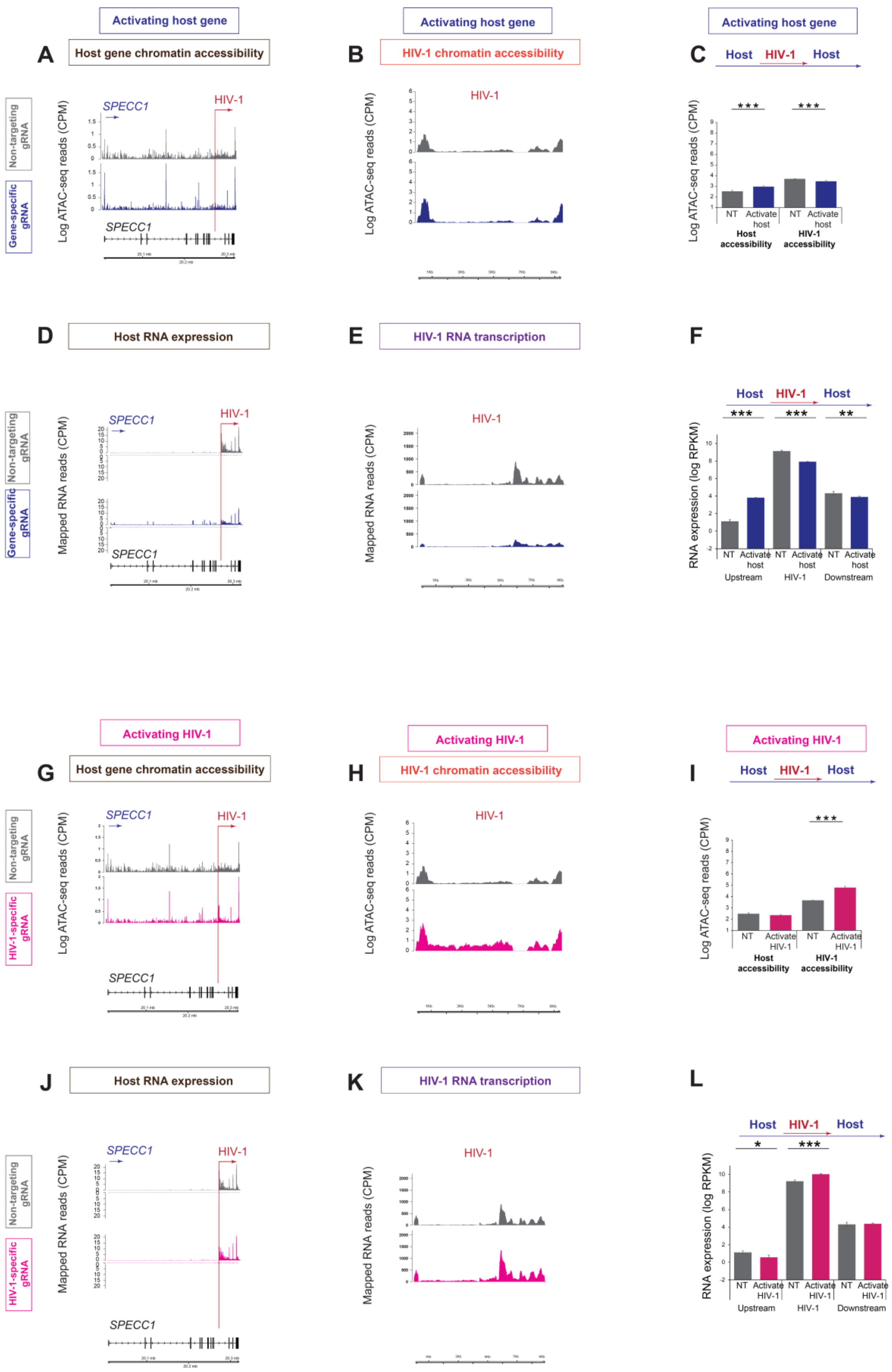

Figure S2. Impact of host versus HIV-1 perturbation on host versus HIV-1 chromatin accessibility in the HIV-1-infected Jurkat T cell clone 1D7 at the *SPECC1* integration site. (**A–C, G–I**) Normalized chromatin accessibility measured by ATAC-seq in this Jurkat T cell clone in which HIV-1 is integrated into host gene *SPECC1* in the same orientation. **(D–F, J–L)**, normalized RNA transcription by RNA-seq. * P < 0.05; ** P < 0.01; *** P < 0.001, Bonferroni corrected, determined by edgeR.

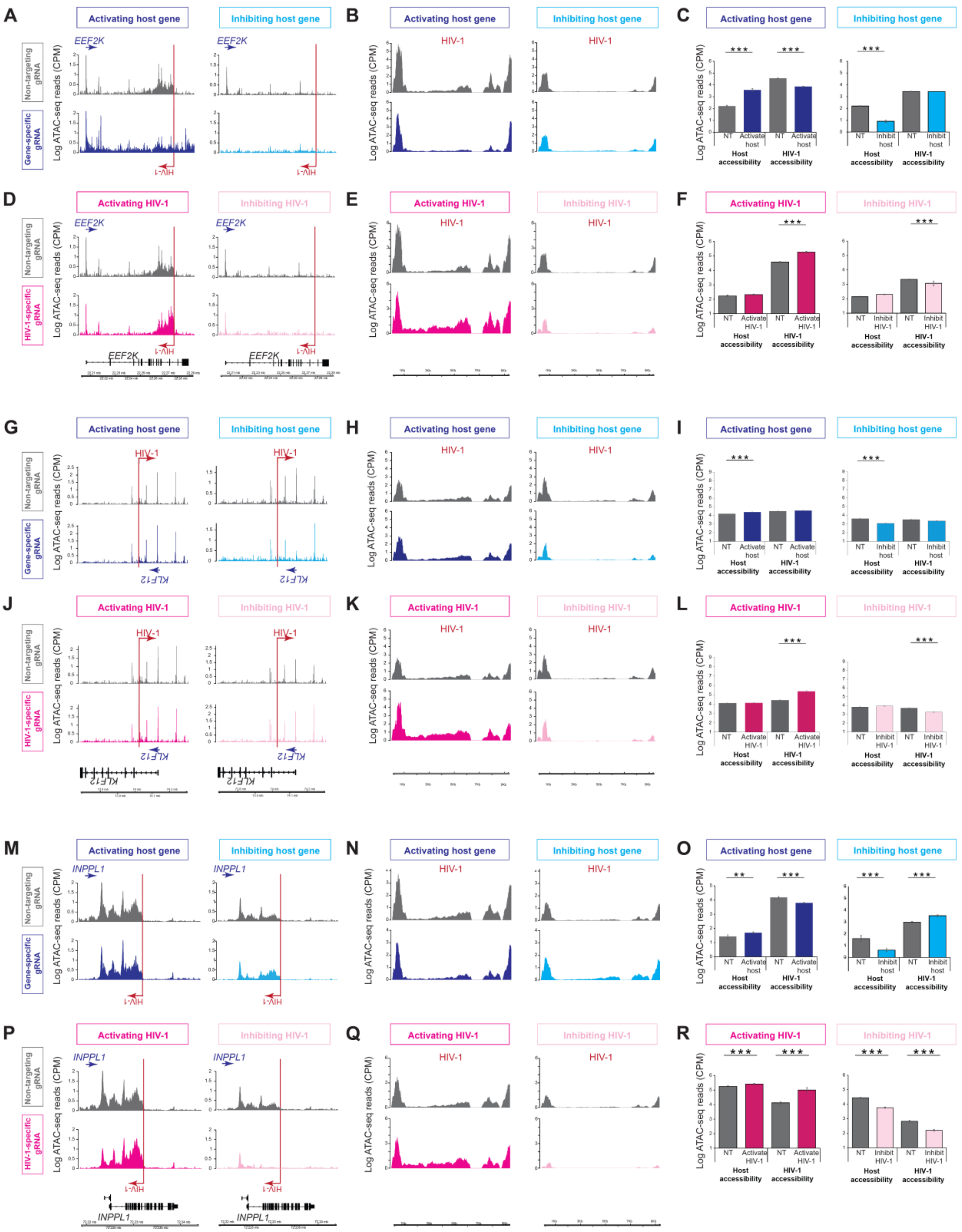

Figure S3. Impact of host versus HIV-1 perturbation on host versus HIV-1 chromatin accessibility when HIV-1 is integrated into host genes in the opposite orientation at the *EEF2K*, *KLF12*, and *INPPL1* integration sites. (**A**–**R**) Host gene and HIV-1 chromatin accessibility upon CRISPR activation versus inhibition of host gene versus HIV-1. Host gene and HIV-1 chromatin accessibility after activation versus inhibition of host gene (**A**–**C**, **G**–**I**, **M**–**O**) and HIV-1 (**D**–**F**, **J**–**L**, **P**–**R**) in Jurkat T cell clones harboring HIV-1 integrated into *EEF2K* (**A**–**F**), *KLF12* (**G**–**L**), and *INPPL1* (**M**–**R**). For ATAC-seq analysis, to account for patterns of chromatin accessibility, peaks within 10 kb were assigned a single Bonferroni-corrected P value equal to the lowest P value among the peaks, divided by the number of peaks within the 10 kb region. Calculated P values were then Bonferroni-adjusted for the six comparisons performed on each sample determined by edgeR. * P < 0.05; ** P < 0.01; *** P < 0.001.

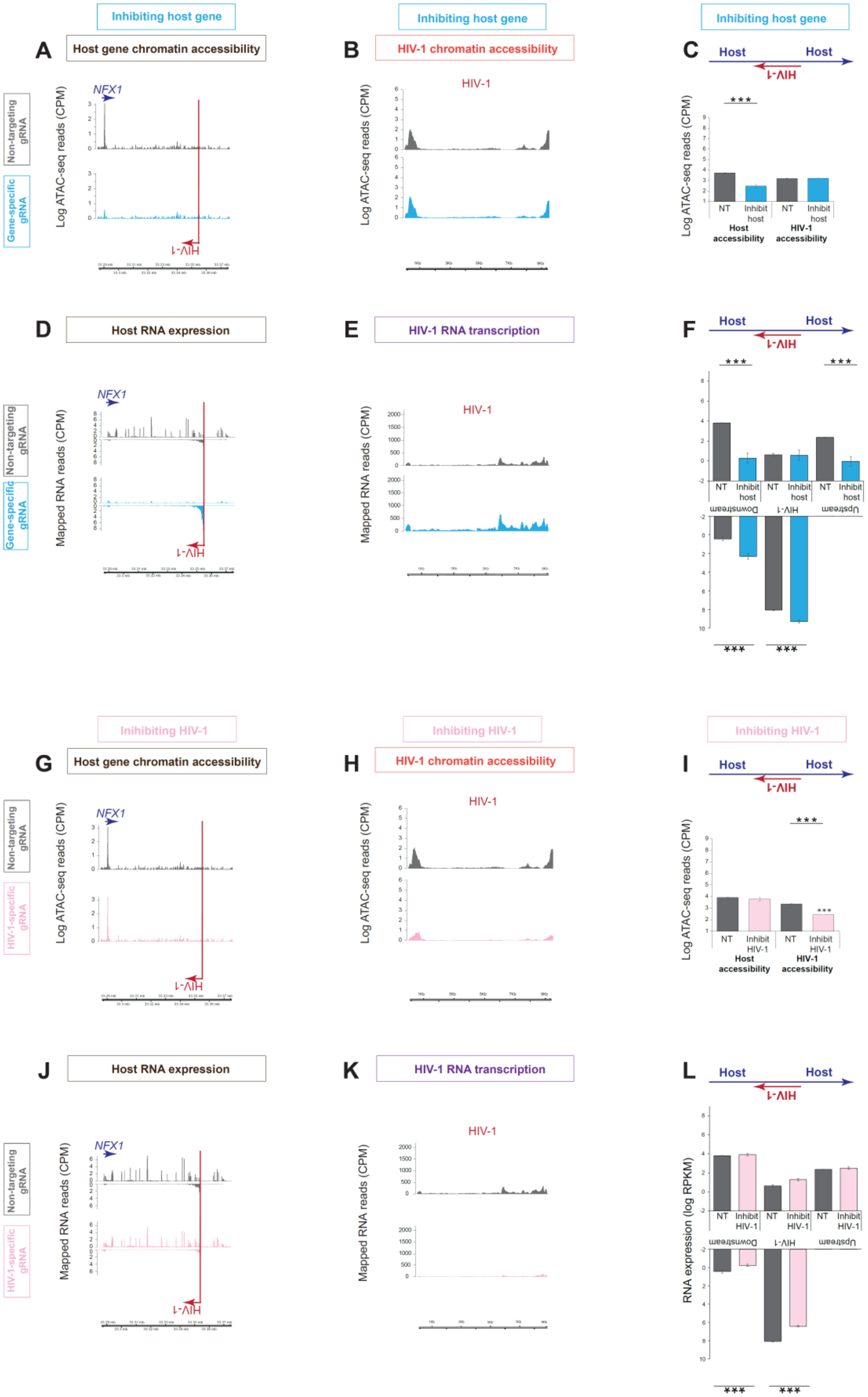

Figure S4. Impact of host versus HIV-1 perturbation on host versus HIV-1 chromatin accessibility in the HIV-1-infected Jurkat T cell clone 8B10 at the *NFX1* integration site. (**A–C, G–I**) Normalized chromatin accessibility measured by ATAC-seq in this Jurkat T cell clone in which HIV-1 is integrated into host gene *NFX1* in the opposite orientation. **(D–F, J–L)**, normalized RNA transcription by RNA-seq. * P < 0.05; ** P < 0.01; *** P < 0.001, Bonferroni corrected, determined by edgeR.
